# An Interactive Website for Whiplash Management (My Whiplash Navigator): Process Evaluation of Design and Implementation

**DOI:** 10.2196/12216

**Published:** 2019-08-26

**Authors:** Aila Nica Bandong, Martin Mackey, Andrew Leaver, Rodney Ingram, Michele Sterling, Carrie Ritchie, Joan Kelly, Trudy Rebbeck

**Affiliations:** 1 Discipline of Physiotherapy Faculty of Health Sciences The University of Sydney Sydney Australia; 2 Department of Physical Therapy College of Allied Medical Professions The University of the Philippines Manila Philippines; 3 Recover Injury Research Centre The University of Queensland Brisbane Australia; 4 Centre of Research Excellence in Road Traffic Injury Recovery The University of Queensland Brisbane Australia; 5 Menzies Health Institute Queensland Griffith University Gold Coast Australia; 6 Kolling Institute John Walsh Centre for Rehabilitation Research The University of Sydney Sydney Australia

**Keywords:** primary health care, whiplash injuries, clinical decision support, clinical pathways, rehabilitation

## Abstract

**Background:**

Whiplash is a health and economic burden worldwide. Contributing to this burden is poor guideline adherence and variable management by health care professionals (HCPs). Web-based tools that facilitate clinical pathways of care are an innovative solution to improve management.

**Objective:**

The study aimed to develop, implement, and evaluate a Web-based tool to support whiplash management following a robust process.

**Methods:**

This study followed the first 3 processes of a research translation framework (idea generation, feasibility, and efficacy) to inform the development, implementation, and evaluation of a website that supports HCPs in whiplash management. Development followed the idea generation and feasibility processes to inform the content, design, features, and functionality of the website. This involved stakeholder (eg, industry partners, website developers, and HCPs) consultations through face-to-face meetings, surveys, and focus group discussions. Implementation followed the feasibility process to determine the practicality of the website for clinical use and the most effective strategy to promote wider uptake. Implementation strategies included classroom education, educational meetings, educational outreach, reminders, and direct phone contact. The analysis of website use and practicality of implementation involved collection of website metrics. Evaluation followed the feasibility and efficacy processes to investigate the acceptability and extent to which the website assisted HCPs in gaining knowledge about whiplash management. Surveys were conducted among student, primary, and specialist HCPs to explore ease of access, use, and satisfaction with the website, as well as self-rated improvements in knowledge of risk assessment, management, and communication between HCPs. Website logs of specialist management decisions (eg, shared care, specialist care, and referred care) were also obtained to determine actual practice.

**Results:**

The development process delivered an interactive, user-friendly, and acceptable website, *My Whiplash Navigator*, tailored to the needs of HCPs. A total of 260 registrations were recorded from June 2016 to March 2018, including 175 student, 65 primary, and 20 specialist HCPs. The most effective implementation strategies were classroom education for students (81% uptake, 175/215) and educational meetings for primary HCPs (43% uptake, 47/110). Popular pages visited included *advice and exercises* and *risk assessment*. Most HCPs agreed that their knowledge about risk management (79/97, 81%) and exercises (85/97, 88%) improved. The specialists’ most common management decision was *shared care*, an improvement from a previous cohort. Areas to improve were navigation and access to outcome measures.

**Conclusions:**

A robust process resulted in an innovative, interactive, user-friendly, and acceptable website, the *My Whiplash Navigator*. Implementation with HCPs was best achieved through classroom education and educational meetings. Evaluation of the website showed improved knowledge and practice to be more consistent with a risk-based clinical care pathway for whiplash. The positive results provide sufficient evidence to scale implementation nationally and involve other target markets such as people with whiplash, insurers, and insurance regulators.

## Introduction

### Background

Whiplash-associated disorders (WAD) remain a huge health and economic burden internationally [1-3], with 50% of people experiencing persistent pain and disability [4-6]. One possible contributor to this burden is that WAD guidelines are inconsistently applied by health care professionals (HCPs) [7,8]. Inconsistent and costly practices include high rates of imaging, excessive use of passive treatments, and delayed and poorly directed specialist referral [8-13]. These practices persist despite extensive and strategic guideline implementation [14-16], highlighting the need for adopting other strategies to promote guideline-based care.

Clinical pathways of care are a promising strategy to improve HCP practice by incorporating guideline recommendations into health care processes [17]. A clinical pathway of care for WAD was developed that uses risk assessment [18,19] and guideline-based treatments matched to the risk of nonrecovery [20]. People at low risk require less intensive treatment and are provided with existing guideline-based resources [21,22]. People at medium and high risk of nonrecovery are referred early to HCPs who specialize in WAD (specialist HCPs), as recommended by WAD guidelines [21]. The specialist HCP more thoroughly assesses the physical and psychological factors associated with nonrecovery. This model of care has been successfully implemented in people with low back pain [23] but is yet to be tested and implemented in people with WAD.

Web-based tools such as computerized decision support systems are one of the most effective strategies to support the implementation of clinical pathways and guidelines, thereby improving HCP practice [24-26]. In musculoskeletal health, these tools are used to provide clinical information, guide triage, and match patients to appropriate resources and treatments [27]. These tools are shown to be effective in improving HCP practice in the management of conditions such as hypertension, diabetes, and osteoarthritis [28,29]. The positive results in other conditions suggest that a Web-based tool might also be an effective strategy to support the WAD clinical pathway of care.

To be effective, Web-based tools need to be developed and implemented using a robust process that ensures maximum uptake by users. Research translation frameworks describe a process from idea generation right through to implementation, evaluation, and then monitoring. For most innovations, such frameworks are followed over decades until an innovation is fully implemented and operational. Although several frameworks exist to guide the process [30], the New South Wales (NSW) Translational Research Framework was applied to develop a suitable Web-based tool for HCPs within the NSW and similar Queensland (QLD) health schemes [31]. Important initial steps of the framework are idea generation, feasibility, and efficacy [31]. Idea generation involves an integrated, multidisciplinary, collaborative approach to develop an innovative intervention [31]. Feasibility determines acceptability and viability of the intervention before further testing and attempts to answer the question of whether the intervention is practical to implement [31]. Implementation strategies known to change practice [14,16,32-35] may be used in this step to determine the practicality of implementing the intervention. During both idea generation and feasibility, stakeholder engagement is crucial to ensure suitability and acceptability of the intervention [31]. Finally, efficacy assesses whether the intervention can deliver expected outcomes under best circumstances. Results of these initial steps gather useful insights to inform further improvements and determine the capacity of the intervention to be successfully scaled up for greatest impact.

Process evaluation is an essential methodology that is used to evaluate the change process at each stage of intervention development and implementation [36,37]. This methodology assesses elements such as reach, effectiveness, and influencing factors to optimize the design and evaluation of the intervention [36,37]. Interventions developed following similar processes have been successful in delivering acceptable, easy-to-use, and effective Web-based tools for conditions such as rheumatoid arthritis [38], obesity [39], and cancer [40].

### Objective

This study used process evaluation methodology to report on the development, implementation, and evaluation of a Web-based tool to support HCPs in the management of WAD.

## Methods

### Design

**Figure 1:**
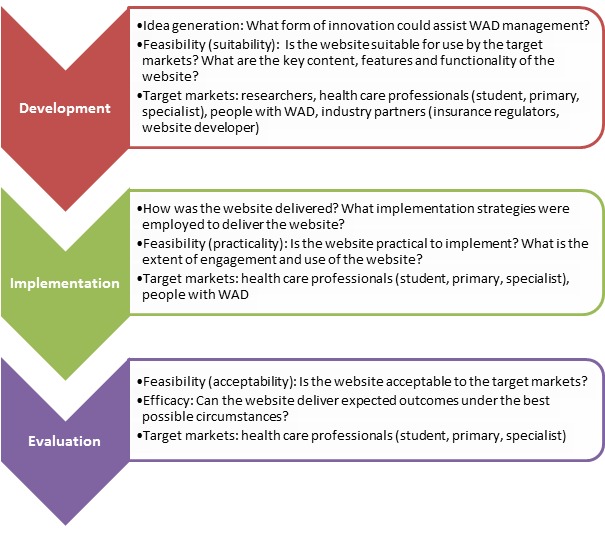
Process diagram summarizing the key stages, research translation process, and target markets to deliver the My Whiplash Navigator website. WAD: whiplash-associated disorder.

This study followed the stages of development, implementation, and evaluation, incorporating the first 3 processes of the NSW Translational Research Framework [31] within each stage (Figure 1). The study involved both quantitative and qualitative methodologies. Ethics approval was provided by the University of Sydney (2015-444) and Griffith University (2015-707) Human Research Ethics Committees.

### Development

The aim of the development stage was to deliver a suitable website to support HCPs in WAD management. During this stage, the idea generation and feasibility processes of the framework were followed.

The idea generation process first involved stakeholder consultation. Initial consultations between researchers from the University of Sydney and University of Queensland identified key stakeholders, including industry partners, primary HCPs, specialist HCPs, and people with WAD. Second, face-to-face meetings were held with industry partners, the compulsory third party (CTP) insurance regulators (ie, NSW State Insurance Regulatory Authority and QLD Motor Accident Insurance Commission). Third, a survey (idea generation questionnaire) was conducted among primary and specialist HCPs and people with WAD to explore their perspectives about existing and ideal WAD resources. Primary and specialist HCPs and people with WAD were invited from our existing research databases. The survey was administered using the Research Electronic Data Capture (REDCap) tool hosted at the University of Sydney [41]. Participants rated their opinions on current resources using a 5-point Likert scale (1=strongly disagree to 5=strongly agree), and data were analyzed using descriptive statistics. The final step involved selecting and then consulting with a vendor who had experience in building interactive health education websites. A transparent tendering process was followed to select a suitable vendor to design the website.

The feasibility process in the development stage involved face-to-face meetings with industry partners and website designers, followed by focus groups discussions among primary and specialist HCPs. A total of 20 work-in-progress meetings were held over 8 months to develop the website, involving 4 policy makers, 8 researchers, 6 clinicians, and 4 website designers. Discussions included design concepts, content, functionality, maintenance and support, milestones, and administration. The researchers had input to the content and sought feedback from key experts during this process. The vendor’s role was to ensure the content was adapted to be suitable for website and target market use.

A total of 6 semistructured focus group discussions were conducted among primary and specialist HCPs to explore perceptions on ideal features, functionality, and content that would facilitate HCP uptake and use. The discussions were run by 2 members of the research team, who were guided by a set of key questions (Multimedia Appendix 1). Proceedings were audio-recorded, transcribed verbatim, and analyzed thematically [42]. Focus group methods have been described in detail elsewhere [43,44].

Before implementation, the website was piloted with 5 HCPs to test the website features and functionality, resolve issues, and further refine the website.

### Implementation

The aim of the implementation stage was to determine the most effective and feasible strategy that could inform future scalable implementation of the website before public release. At this stage, the feasibility process of the framework was followed to also evaluate the practicality of the website. Target markets for this stage were students and primary and specialist HCPs, ensuring a range of clinical experience from novice to experienced HCPs.

Student HCPs were engaged through classroom education. The website was integrated as a learning resource in a unit of study for physiotherapy students at the University of Sydney (n=215). Key components of the educational module included standard assessment for WAD, risk assessment, provision of treatments for people with WAD at low risk of nonrecovery, and timely and appropriate referral to specialist HCPs for people with WAD at medium and high risk of nonrecovery.

Primary HCPs were engaged through educational courses, educational outreach, and reminders. We targeted 3 professional development courses in WAD that were conducted by the Australian Physiotherapy Association over a 12-month period. A total of 110 physiotherapists attended these courses. The key features and components of the website were explained and integrated into the WAD educational modules. In addition, the website was promoted through 4 conference presentations. Primary HCPs who attended the courses and consented to participate were followed up with educational outreach, either face-to-face or over the phone, and provided with standard educational packages. In addition, reminders were sent through social media (eg, Facebook) and regular newsletters.

Specialist HCPs (n=20) were engaged through 2 targeted educational workshops. The content of the first educational workshop was based on results of a qualitative study, focusing on the role of specialist HCPs, management decisions for people with WAD at medium and high risk of nonrecovery, and effective communication with primary HCPs [43]. Key features and use of the website that facilitate the above were also discussed during the educational workshop. A follow-up face-to-face meeting was conducted after 6 months to address issues such as access to certain resources and documentation of management decisions.

Analysis of website use and the practicality of website implementation involved assessment of reach and extent of engagement by HCPs. Website traffic and trends (eg, registered users, total visits, total page views, and top landing pages) were collected through Google analytics and built-in website reports.

### Evaluation

The aim of the evaluation stage was to investigate acceptability and preliminary efficacy of the website among HCPs. The framework processes followed were feasibility and efficacy. This was achieved by inviting all HCPs registered on the website to complete an evaluation questionnaire and determining actual practice through website logs.

The feasibility process in the evaluation stage assessed acceptability of the website by students and primary and specialist HCPs. The evaluation questionnaire asked questions regarding ease of access, use, and satisfaction with the website. At least 10 website users were necessary to obtain enough data for evaluation. This number was determined a priori and based on available evidence and previous studies that explored usability of Web-based resources for management of other conditions [38,45,46].

Efficacy was evaluated by measuring the extent to which the website assisted HCPs in gaining knowledge of key components of WAD management. Students and primary and specialist HCPs were asked about their self-rated improvement in knowledge of risk assessment and management of people with WAD at low risk and those at medium and high risk of nonrecovery. Furthermore, primary and specialist HCPs were asked about their opinion whether the website facilitated communication between HCPs. This item was specifically asked to primary and specialist HCPs, given their access to the website feature that allows documentation of management decisions in patient care. Actual practice was documented by reviewing website logs of decisions made by specialist HCPs.

Close-ended questions were rated using a 5-point Likert scale (1=strongly disagree to 5=strongly agree). Open-ended questions explored opinions on the best features of the website, barriers to use, and suggestions for further improvement. Survey data were analyzed using descriptive statistics, and responses to open-ended questions were grouped into common categories.

## Results

### Development

The stakeholder consultation generated ideas that informed website design and implementation. Key features and content of the website were identified successively as a result of the consultation process.

Researchers and CTP insurance regulators determined that the website should assess the risk of nonrecovery and facilitate risk-based management. The clinical prediction rule (CPR) developed by researchers from the University of Queensland [18,19] was agreed to be used and automated on the website. Concurrently, consultation between researchers from the University of Sydney and CTP insurance regulators had developed a decision matrix for specialist HCPs to assist in the management of people with WAD at medium and high risk of nonrecovery. The decision matrix included 3 management decisions: (1) shared care (ie, continued treatment with the primary HCP monitored by a specialist), (2) specialist care (ie, direct treatment from specialist), and (3) referred care (ie, referral to other disciplines).

Researchers and CTP insurance regulators further identified that the website should consolidate evidence-based and risk-based resources for people with WAD and HCPs. Before the development of the website, patient versions of the WAD guidelines, educational and exercise videos, factsheets, and booklets were previously developed by the CTP insurance regulators with extensive involvement of people with WAD. Resources for HCPs who manage people at low risk were based on the most recent WAD guidelines and were available in various formats (eg, booklets, factsheets, and videos) [21,22]. Resources for HCPs who manage people at medium and high risk were to facilitate referral to specialist HCPs and assist in further assessment of physical and psychological factors.

The idea generation survey was returned by 94 HCPs (94/671, 14.0%) and 26 people with WAD (26/50, 52%). About half of the HCPs (53/94, 56%) and most of the people with WAD (25/26, 96%) surveyed had not seen the existing WAD resources. When these resources were shown to them, most HCPs (59/94, 63%) and people with WAD (21/26, 81%) agreed that these would help WAD management and applied to their injury (Table 1). In addition, the majority of the people with WAD who completed the survey agreed that they would appreciate access to the resources. About half of the HCPs (50/94, 53%) also indicated that a website would be the best platform to facilitate access to the resources.

Results of the focus group discussions, attended by 28 HCPs (16 primary and 12 specialist HCPs), endorsed some key features of the website and informed the types of resources that were added to the website (Multimedia Appendix 2). Themes that were generated can be broadly categorized as those relating to risk assessment, management of people at low risk of nonrecovery, and management of people at medium and high risk of nonrecovery. For example, HCPs suggested that if risk assessment was to be automated on the website, then guidance on what to say to people at different risk levels should be provided. In the low risk section of the website, primary HCPs suggested downloadable and customizable exercise sheets be provided. In the high-risk section of the website, primary HCPs indicated that a database of specialist HCPs incorporated into the website would facilitate the referral process. Finally, specialist HCPs suggested accessible and downloadable psychological screening tools (eg, Depression, Anxiety and Stress Scale [47], and Pain Catastrophizing Scale [48]) and other outcome measures (eg, Self-report Leeds Assessment of Neuropathic Symptoms and Signs pain scale [49], and Central Sensitization Inventory [50]) as well as incorporating a mechanism to document management decisions and communicate with primary HCPs. These results were considered, and the website incorporated content that provided suggestions on communicating risk level, downloadable resources (eg, screening tools, exercise sheets, and outcome measures) and a database of specialist HCPs.

**Figure 2:**
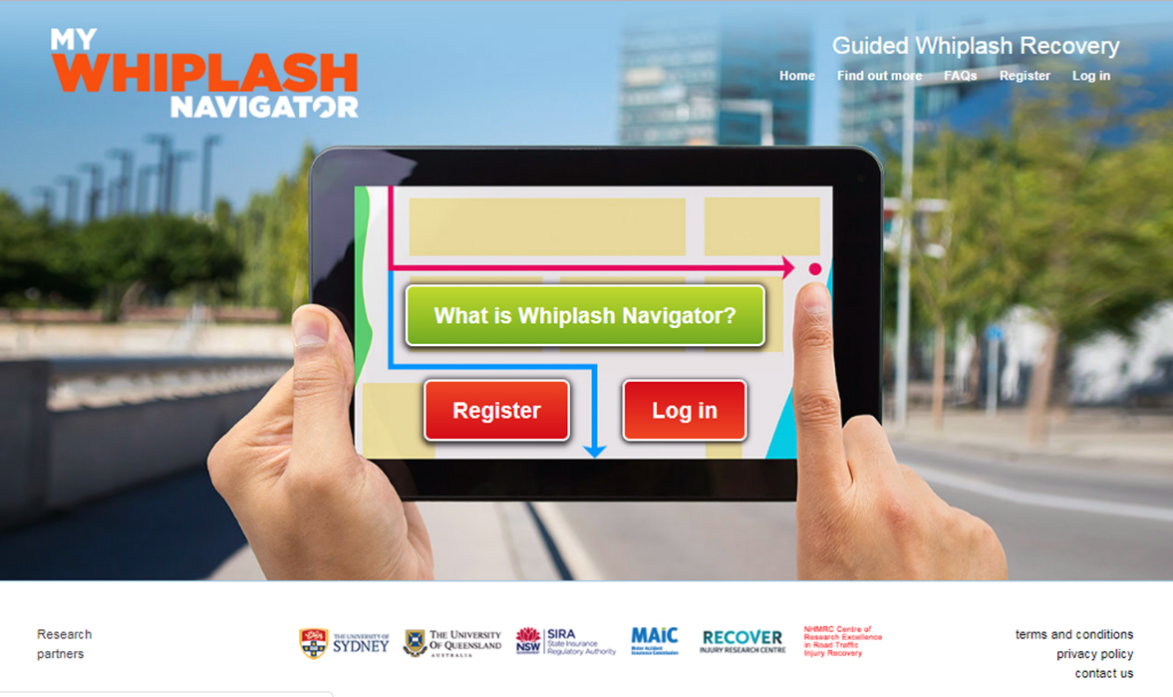
The *My Whiplash Navigator* website landing page.

The vendor consultation resulted in the delivery of an interactive, user-friendly website, the *My Whiplash Navigator* (Figure 2). Within the website, 3 sections were developed: patient, health care practitioner, and specialist practitioner. Each section was developed with the target market considered and the key components of WAD management embedded (ie, risk assessment and risk-based management; Multimedia Appendix 3). The patient section guides people with WAD through various steps within the clinical pathway, including risk assessment, and provides access to useful information, advice, and exercises. The health care practitioner section comprises guideline-based resources to assist in WAD management, particularly management of people at low risk and referral of those at medium and high risk of nonrecovery. The specialist practitioner section was designed to assist the specialist in making appropriate decisions for people who are at medium and high risk of nonrecovery.

**Table 1:** Perceptions of health care professionals (N=94) and people with whiplash-associated disorder (N=26) about available resources on whiplash before the project.

Target group			Strongly disagree/disagree, n (%)	Neutral, n (%)	Strongly agree/agree, n (%)
**Health care professionals**					
	**Factsheet**				
		Helpful in WAD^a^ management	12 (13)	26 (28)	56 (60)
		Would use the resource in practice	19 (20)	20 (21)	55 (59)
	**Educational video**				
		Helpful in WAD management	9 (10)	23 (25)	62 (66)
		Would use the resource in practice	22 (23)	26 (28)	46 (49)
	**Exercise video**				
		Helpful in WAD management	12 (13)	22 (23)	60 (64)
		Would use the resource in practice	24 (26)	25 (27)	45 (48)
**People with WAD**					
	**Factsheet**				
		Applicable to my injury	1 (4)	3 (12)	21 (84)
		Would want access to the resource	1 (4)	1 (4)	23 (92)
	**Educational video**				
		Applicable to my injury	0 (0)	5 (19)	20 (80)
		Would want access to the resource	1 (4)	4 (15)	20 (80)
	**Exercise video**				
		Applicable to my injury	0 (0)	5 (19)	20 (80)
		Would want access to the resource	2 (8)	4 (15)	19 (76)

^a^WAD: whiplash-associated disorder.

#### Format, Layout, and Features

**Figure 3:**
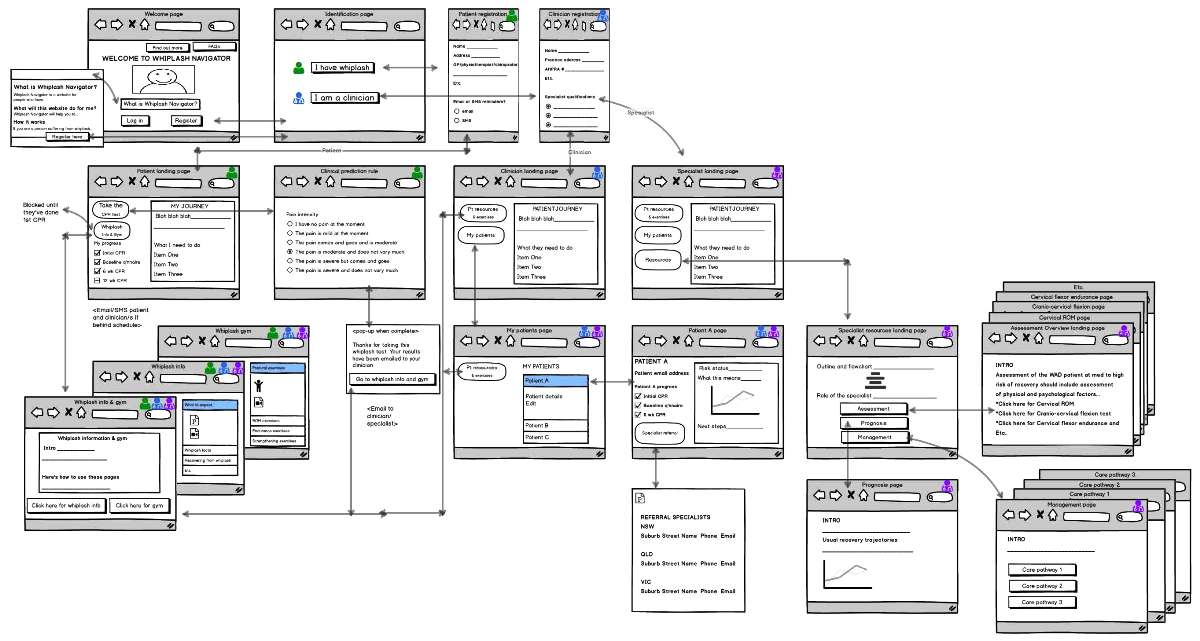
The *My Whiplash Navigator* wireframes overview.

The content of the website was written in a professional and patient-friendly tone, included interactive and intuitive pages, and presented a positive and encouraging color scheme and layout. Pages were interactive, intuitive, and user friendly to facilitate ease of navigation. Information contained within the website were presented as navigation links and in a drop-down/accordion format, where appropriate. The interactions and access to various pages within the website are summarized in Figure 3.

The homepage of the website provided information about the *My Whiplash Navigator* and links to register and capture login details. Once registered or logged in, the patient or HCP is directed to their relevant page. The patient section includes an automated version of the whiplash CPR. The patient completes a questionnaire, and the score/risk status is computed. Feedback about the level of risk is provided to the patient and the treating HCP, with a link to information for matched treatments according to the risk of nonrecovery. Suggestions on how to communicate risk level to patients are provided to the treating HCP. A database of specialist HCPs is also incorporated on the website to facilitate referral of people with WAD at medium and high risk of nonrecovery. In addition, the website links the patient with the treating HCP, as well as the specialist HCP, allowing access to information and monitoring of recovery. Communication between the treating HCP and patient is facilitated through a comments section in the management page. Communication between the treating and specialist HCPs is further facilitated in the management page, where the specialist HCP logs the decisions made and advice for the treating HCP. The management section also allows the specialist HCP to upload relevant documents or reports that the treating HCP is able to view. Resources for patients and HCPs are downloadable, and a customizable exercise dosage chart is also available for HCPs to download.

#### Permissions

The website was built on a Web platform called Drupal, which is a proven, secure application framework that has role-based permissions system. A tiered permission system was used for the *My Whiplash Navigator*. First-tier included *administrators* (eg, researchers and research assistants) who were able to access all information on the website. Other tiers include *patients* and *HCPs*. Patients were tagged as belonging to a particular HCP. Only those HCPs with a tagged relationship with a patient were able to review all the data on that particular patient. No facility exists for them to access other patient data, except through aggregated and anonymized data reports. No patient is able to access another patient’s data. The data are stored on a MySQL (Oracle Corporation) server cluster that is run by a secure Australian data center hosted in Sydney. The physical location for this server has 24/7 on-site security and is ISO27001:2005 certified.

### Implementation

Implementation of the website took place primarily in NSW, QLD, and Australian Capital Territory from June 2016 to March 2018. A total of 260 HCP registrations were recorded (Table 2). The majority of the registrations were from NSW (214/260, 82.3%) followed by QLD (26/260, 10.0%).

A total of 65 primary HCPs registered on the website, with most registrations recorded following educational meetings (47/110, 42.7%). All specialist HCPs engaged through educational workshops registered on the website (20/20, 100%). The majority of the student HCPs registered on the website (175/215, 81.4%) following classroom education.

**Table 2:** Summary of registrations on the *My Whiplash Navigator* website.

Target group	Implementation strategy	Total approached, N	Total registered on the website, n (%)
Student HCP^a^	Classroom education	215	175 (81.4)
Primary HCP	Educational meeting	110	47 (42.7)
	Educational outreach and reminders	51	18 (35)
Specialist HCP	Educational workshops	20	20 (100)

^a^HCP: health care professional.

**Table 3:** Summary of the number and duration of page views for the *My Whiplash Navigator* website.

Page title		Unique page views, N	Average time (seconds)
**Assessment of risk**			
	Health professional assessment of a whiplash injury	280	160.94
	Prognosis: identifying people at risk of poor recovery	200	128.46
**Provision of appropriate management for people at low risk of nonrecovery**			
	Whiplash information and exercises	339	145.48
	Whiplash exercises	192	260.59
	Helpful facts and advice	102	161.99
**Provision of appropriate management for people at medium and high risk of nonrecovery**			
	Specialist assessment of whiplash	94	143.38
	Specialist care for whiplash	84	66
**Other frequently visited pages**			
	*Whiplash Navigator* steps	375	83.68
	Managing whiplash in your patients	217	199.23
	What is *Whiplash Navigator*?	169	91.7
	FAQs^a^ for patients	88	119.31

^a^FAQs: frequently asked questions.

The website pages most commonly viewed were related to risk assessment, exercises, and information about WAD (Table 3). There were 1229 times when users were actively engaged with the website. The majority of website engagement was from new visitors (858/1229, 69.81%), and 30.19% (371/1229) were returning visitors. The total page views of the website were 7508 views, of which 5027 were unique page views. Users view, on average, 6 pages during a visit and stay approximately 3 minutes on each page.

### Evaluation

The website evaluation survey was administered after the implementation stage, between April and June 2018, and returned by 24 primary HCPs (24/65, 37%), 13 specialist HCPs (13/20, 65%), and 60 student HCPs (60/175, 34.3%).

Most HCPs agreed that the website was accessible, easy to navigate, and was a useful resource to assist in WAD management (Table 4). Posthoc analyses showed that there was no difference among students and primary and specialist HCPs regarding the acceptability of the website (Table 5). Most HCPs agreed that they would use the website in their clinical practice (82/97, 85%). Furthermore, the majority of the primary and specialist HCPs would recommend the use of suitable Web-based tools to other practitioners (32/37, 87%). The best features identified were accessibility of the website, simple interface promoting ease of use and navigation, and capacity to link people with WAD with HCPs. Many perceived that the website was comprehensive and provided a *one stop shop* for HCPs.

In contrast, features that needed improvement were initial navigation difficulties and complicated registration process. A few HCPs suggested potential limitation of the reach of the website for HCPs or people with WAD who do not have access to technology. Accordingly, HCPs identified a number of additional features and content to further improve the website. Some HCPs suggested adding more exercise and assessment videos that can be used to further assist patient care. Features such as a search bar, site navigator, and information for first time users would further enhance navigation. Finally, some HCPs wanted to be able to customize exercises and resubmit forms within the website to make monitoring progress and designing exercise programs easier.

**Table 4:** Perceptions of health care professionals about acceptability and self-rated improvement in knowledge of whiplash-associated disorder management after using the *My Whiplash Navigator* website (N=97).

Outcome		Strongly disagree/disagree, n (%)	Neutral, n (%)	Strongly agree/agree, n (%)
**Acceptability of the website**				
	Easy to access screening tools	5 (5)	20 (21)	72 (74)
	Easy to access outcome measures	3 (3)	21 (22)	73 (75)
	Easy to navigate	1 (1)	28 (29)	68 (70)
	Easy to understand	0 (0)	13 (13)	84 (87)
	Useful resource for WAD^a^	0 (0)	11 (11)	86 (89)
	Will use the website in clinical practice	1 (1)	14 (14)	82 (85)
**Self-rated improvement in knowledge**				
	Risk assessment	0 (0)	18 (19)	79 (81)
	Using the C-Spine rule^b^	1 (1)	14 (17)	69 (82)
	Standard assessment^b^	1 (1)	12 (14)	71 (85)
	Risk-based advice	0 (0)	18 (19)	79 (81)
	Appropriate exercises	1 (1)	11 (11)	85 (88)
	Referral of high-risk patients^b^	1 (1)	13 (16)	70 (83)

^a^WAD: whiplash-associated disorder.

^b^n=84.

**Table 5:** Posthoc analyses of differences among student, primary, and specialist health care professionals regarding acceptability and self-rated improvements in knowledge of whiplash-associated disorder management after using the *My Whiplash Navigator* website.

Outcome		Difference between groups^a^, chi-square (df)	Significance, *P* value
**Acceptability of the website**			
	Easy to access screening tools	4.2 (2)	.12
	Easy to access outcome measures	1.8 (2)	.41
	Easy to navigate	1.8 (2)	.41
	Easy to understand	0.6 (2)	.74
	Useful resource for WAD^b^	0.6 (2)	.71
	Will use the website in clinical practice	1.0 (2)	.59
**Self-rated improvement in knowledge**			
	Risk assessment	0.7 (2)	.67
	Risk-based advice	0.0 (2)	.99
	Appropriate exercises	0.6 (2)	.74

^a^Test statistic: Kruskal-Wallis test.

^b^whiplash-associated disorder.

Most HCPs agreed that the website helped them gain knowledge about key aspects of WAD management (Table 4). The highest self-rated improvement was around provision of appropriate exercises (85/97, 88%), whereas the lowest was in the identification of risk of nonrecovery and provision of risk-based advice (79/97, 81%). Similarly, the majority of the specialist HCPs agreed that the website assisted in gaining knowledge about management decisions for people with WAD at medium and high risk of nonrecovery (11/13, 85%). Posthoc analyses demonstrated that there was no difference among student, primary HCPs, and specialist HCPs regarding self-rated improvements in knowledge (Table 5). The majority of the primary and specialist HCPs agreed that the website facilitated communication between the primary health care provider and specialist (25/37, 68%).

In terms of actual practice, there were a total of 24 recorded management decisions logged by specialist HCPs on the website. The most commonly chosen pathway decision was shared care (15/24, 63%). Provision of specialist care was chosen by specialist HCPs in 33% of the decisions logged (8/24), mainly because the people with WAD that they saw did not have primary HCPs at the time of consultation.

## Discussion

### Principal Findings and Comparison With Previous Work

Results of this study demonstrate that the *My Whiplash Navigator* is innovative, practical to implement, acceptable, and assisted in improving knowledge and practice of HCPs in WAD management. The rigorous process of development, implementation, and evaluation involving extensive consultations among stakeholders has led to the first Web-based tool for WAD that puts together comprehensive, evidence-based resources, automated risk assessment, and risk-based management. Stakeholder consultation from the outset is key in research translation [31,51], and incorporating the suggestions of each target market during idea generation and feasibility supported uptake and acceptability of the website. These results demonstrate that the *My Whiplash Navigator* may be scaled for wider implementation.

Most of the development and implementation strategies used for this website have succeeded in engaging the target markets and can be used in a future wider implementation program. Known implementation strategies such as classroom education [52,53] and educational meetings [14,16,32] used to improve practice in other conditions were also effective in engaging HCPs to use the website. Incorporating the website as part of assessable educational content further enhanced implementation, given higher uptake among student HCPs compared with primary HCPs. Second, an effective and efficient way to facilitate website use among people with WAD might be through primary HCPs and insurers. Compared with the fast-paced, high-stress environment in hospital emergency departments [54], the typical clinical encounter and the therapeutic relationship between people with WAD and their primary HCPs (Griffin et al, under review) would more likely assist in engaging people with WAD to use the website. Primary HCPs are also the first point of contact of people with WAD, with general practitioners consulted as early as 4 days and physiotherapists at 3 weeks after injury [8]. Finally, insurance regulators are equally in a position to engage people with WAD because most people who get injured from motor vehicle crashes in NSW and QLD submit claims after the accident to access benefits under the CTP insurance scheme.

Results also showed that implementation of the website improved HCP knowledge and practice across the health service delivery spectrum (ie, from novice to experienced HCPs) that could potentially improve outcomes for people with WAD. Preliminary efficacy demonstrated that the website improved self-rated knowledge of HCPs about key aspects of WAD management, specifically, self-rated knowledge of risk assessment and provision of risk-based advice. Although previous studies have shown that it has been difficult to improve knowledge on risk assessment among primary HCPs [14,16,32], use of the website appeared to support the delivery and implementation of these key messages. Similarly, use of the website appeared to improve actual practice of specialist HCPs through the promotion of a shared care approach in managing people with WAD at medium and high risk of nonrecovery. This is in contrast to the results of a previous study where the same group of specialist HCPs preferred to provide direct treatment (60%) over shared care (22%) [43]. A shared care approach in management was perceived by primary HCPs to facilitate the referral process and recovery for people with WAD [43]. The strategies used to engage specialist HCPs along with the interactivity of the website may have contributed to the improvements in practice observed.

### Next Steps

The positive results related to the feasibility and preliminary efficacy of the website demonstrated the potential for broader uptake and implementation. Results further identified website content and features that need to be improved before more widespread implementation. The next process in translation would be that of replicability and scalability [31]. Replicability involves testing whether the website could deliver the same outcomes in other circumstances. Ideally, the aim is to implement the website in other states in Australia to test its application for WAD in different motor accident CTP insurance schemes. In addition, implementation of the website could involve other key target markets such as people with WAD, the insurance industry, and HCPs from other disciplines involved in WAD management (eg, chiropractors, osteopaths, and general practitioners). Finally, the suggestions from HCPs to incorporate additional features such as search or navigator bar and access to outcome measures would be considered to facilitate easier navigation and better clinical decision making.

### Limitations

The response rate for the idea generation survey among HCPs was low; however, the survey was meant to be exploratory to supplement the other processes used during the development stage of the website. Despite a low response rate, the responses received from HCPs captured divergent opinions allowing us to understand the needs and expectations related to resources to assist HCPs in WAD management. People with WAD were not included in the implementation and evaluation stage, mostly because access to the website was limited to patients enrolled in a concurrent randomized controlled trial (RCT) [20] that the website supports. However, before this work, people with WAD were extensively involved in the development of the patient version of the WAD guidelines, education and exercise videos, factsheets, and booklets for people with WAD. These resources formed the patient section of the website. Implementation and evaluation of the website among people with WAD will be conducted and reported on after completion of the RCT.

### Conclusions

In summary, a robust process resulted in an innovative, interactive, user-friendly website, the *My Whiplash Navigator*. Implementation with HCPs was best achieved through classroom education and educational meetings. Evaluation suggested that the website was acceptable and improved knowledge and practice of HCPs in WAD management. These positive results provide sufficient evidence to scale implementation nationally and involve other target markets such as people with WAD, insurers, and insurance regulators.

## Appendix

Multimedia Appendix 1 Focus group discussion questions for health care professionals during the idea generation process of website development.

Multimedia Appendix 2 Summary of themes, codes, and illustrative quotes from the focus group discussions.

Multimedia Appendix 3 Design features, content, and functionality of key pages of the *My Whiplash Navigator* website.

## Abbreviations

CPR: clinical prediction rule
CTP: compulsory third party
HCP: health care professional
NSW: New South Wales
QLD: Queensland
RCT: randomized controlled trial
WAD: whiplash-associated disorder
