# Supplementary material for: An Interactive Website for Whiplash Management (My Whiplash Navigator): Process Evaluation of Design and Implementation
Source: JMIR Form Res. 2019 Aug 26;3(3):e12216. doi: 10.2196/12216 (PMC6732967; doi:10.2196/12216)
Supplement: Multimedia Appendix 1 [file formative_v3i3e12216_app1.pdf]

**Multimedia Appendix 1.** Focus group discussion questions for health care professionals during the idea generation process of website development.

| Stimulus question | Prompts |
|---|---|
| 1. How do you think the Whiplash CPR could be best implemented in practice? | • What format would be most appropriate and accessible?<br>• What information would assist you in conveying the meaning of each categorisation (likely recovery etc.) to a patient? |
| 2. What other kinds of resources would be useful in the management of patients with WAD? | • For you as primary care practitioners/specialists?<br>• For patients? |
| 3. How do you think the resources associated with these guidelines could be best organised on a website for primary carers? | • What format would be most appropriate and accessible?<br>• What do you need that is not there?<br>• How should the exercises be best categorized?<br>• What is your opinion about the suggested website format for these resources? |
| 4. [Specific question to specialists] What resources would be useful to perform your role as specialists (and those training to become specialists)? | • What resources would be most useful when choosing your management decision?<br>• What format would these resources be best organised?<br>• What do you think about using case vignettes, audio/video clips (e.g. liaising with a primary care provider) as a means to assist with choosing management decisions? |

[a]WAD: whiplash associated disorder.
