# Supplementary material for: An Interactive Website for Whiplash Management (My Whiplash Navigator): Process Evaluation of Design and Implementation
Source: JMIR Form Res. 2019 Aug 26;3(3):e12216. doi: 10.2196/12216 (PMC6732967; doi:10.2196/12216)
Supplement: Multimedia Appendix 2 [file formative_v3i3e12216_app2.pdf]

Multimedia Appendix 2. Summary of themes, codes and illustrative quotes from the focus group discussions.

Themes	Codes	Illustrative quotes
Risk assessment	Automated screening	P3: … I would like to see a system where I get given like a portal access… generate a link… and they [patients] can complete it on their iPad, phone, or their PC and the results will automatically come back to me…<br><br>P22: If it's simple calculator, we put the data in… and it calculates… and you collate the data points yourself.<br><br>P20: I think having resources there where it could score it for you and say "This person is low risk or high risk, whatever."
	Guidance to communicate risk to patients	P4: I think the wording of the results [risk assessment] will have to be very careful… We have to make sure there's not a massive label.<br><br>P7: It's always a balance between keeping them informed and keeping them positive…<br><br>P8: I think the first message is don't tell them which category they fall under... it's only going to promote negative thinking… not going to help in their recovery.<br><br>P22: I think de-catastrophising, education around pain, making it simple… There's going to be some acknowledgement of the injury…
Management of people at low risk of non-recovery	Downloadable and customisable exercise sheets	P9: The practitioner makes a selection of the exercises and then prints it off and emails it to them.<br><br>P14: You've got space to personalise it… you've got access to change that [exercises]…<br><br>P16: The ability to pick and customize the exercises for them [patients] relevant to their case, not general.<br><br>P28: If you've got something like this [exercise sheets] that you can individualise for your patient, then I think that's a good resource to have.
	Accessible resources	P1: … You [practitioner] are directing them [patients] to a reliable source or link of information rather than have them google.<br><br>P3: A point or a portal where you can actually have the latest evidence-based exercises.<br><br>P28: It probably needs to be [designed such that] whatever they're looking for they can get that… specifically for the patient… specifically for the practitioner… so they can easily find that information
	Additional resources	P2: A video to be able to look at online just demonstrating the exercises perhaps.<br><br>P5: Some recommended sentences… if we have something more structured for reassurance so that people are provided reassurance not just being casual about it.

		P9: Just on the website, if you can [put] things in there that promote basically self-management, self-efficacy...<br>P15: It's important to have some red flags… to know if they're [patients] experiencing anything with those exercises.<br>P16: … Webpage with different exercises listed there and then you can just click on it<br>P17: … A lay description of what sort of injury people sustain, just so they [patients] understand that it's your general and acute soft tissue injury…<br>P19: Maybe other information part… tips and some general information [about whiplash].<br>P22: In terms of resources… brief, 15-minute, 10-minute instructional video.<br>P23: … An automatic direct line, that automatically directs to that video.
Management of people at medium/high risk of non-recovery	Database of WAD[a] specialists	P4: Database of who and where they are.<br>P5: List of specialists in the area.<br>P8: A physical barrier would just be where to send them. If on the website, you got a list of people, so then you got the confidence to say… they're [specialist HCPs] the right person."<br>P19: I think a list of who to refer to will be really nice, like a nice contact...
	Information on complex assessments	P20: Anxiety and depression scale… You had one about pressure pain thresholds…<br>P22: How to screen psychologically without doing a battery of tests, a battery of papers… What are the key physical parameters…<br>P21: Cut-off scores… What the scores of the different questionnaires mean. What is an acceptable clinical change too… It's the content as well aside from the score.<br>P26: I guess your qualitative sensory testing… what that actually looks like in a clinical sense.
	Case vignettes	P26: Could you have clinical cases online? … I think that would give someone a good enough idea of how to seamlessly put an examination together.<br>P18: … Vignettes- they are a useful tool but they certainly provide you with anywhere near the same information as actually seeing the patient…<br>P23: Case vignettes with supporting literature… and then there's an outcome, an actual competency evaluation at the end… So it's actually more than just ticking the box.

[a]WAD: whiplash associated disorder.
