# Supplementary material for: An Interactive Website for Whiplash Management (My Whiplash Navigator): Process Evaluation of Design and Implementation
Source: JMIR Form Res. 2019 Aug 26;3(3):e12216. doi: 10.2196/12216 (PMC6732967; doi:10.2196/12216)
Supplement: Multimedia Appendix 3 [file formative_v3i3e12216_app3.pdf]

Multimedia Appendix 3. Design features, content and functionality of key pages of the *My Whiplash Navigator* website.

Page	Content, features and functionality	Sample content
Patient page	• online version of the whiplash clinical prediction rule<br>• feedback regarding risk of non-recovery<br>• patient-specific resources (e.g. information, advice and exercises)<br>• videos and images of exercises and downloadable information<br>• tracking of the patient's progress through completion of questionnaires	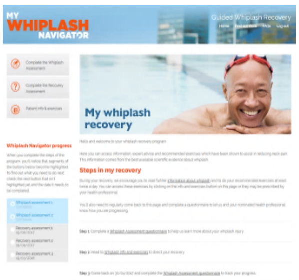
Primary healthcare provider page	• feedback to the primary healthcare provider about the patient's risk classification<br>• suggestions for communicating risk of non-recovery to the patient<br>• resources on assessment, prognosis, advice and exercises to patients, and referral to specialists<br>• downloadable and customisable exercise chart<br>• ability to view and download management plans or patient care/management decisions made by the primary healthcare provider and specialist physiotherapist<br>• schedule for review of progress<br>• link to patient and specialist physiotherapist<br>• database of specialist physiotherapists in two NSW[a] and QLD[b]	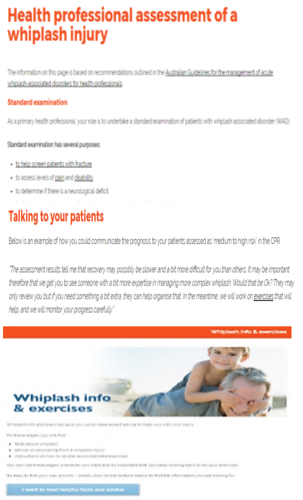
Specialist provider page	• access to guideline-based resources on assessment, prognosis, advice and exercises to patients, and referral to specialists (SIRA[c], 2014)<br>• information about more complex assessments, appropriate medications, and evidence-based treatments<br>• ability to view and download management plans or patient care/management decisions made by the primary healthcare provider and specialist physiotherapist<br>• interactive case vignettes to illustrate the three decision tasks of specialists: shared care, specialist care, and referral for alternate care<br>• link to primary healthcare provider and patient	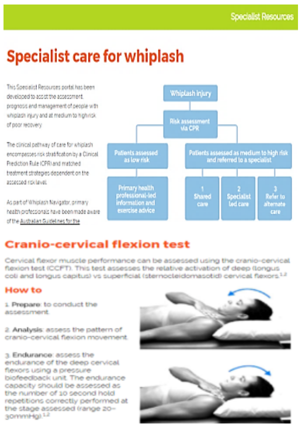

[a]NSW: New South Wales.

[b]QLD: Queensland.

[c]SIRA: State Insurance Regulatory Authority.
